# A Canadian Prospective Study of Linkage of Randomized Clinical Trial to Cancer and Mortality Registry Data

**DOI:** 10.3390/curroncol28020111

**Published:** 2021-03-08

**Authors:** Annette E Hay, Nicole Mittmann, Michael Crump, Matthew C Cheung, Jessica Sleeth, Judy Needham, Mike Broekhoven, Marina Djurfeldt, Lois E Shepherd, Ralph M Meyer, Bingshu E Chen, Joseph L Pater

**Affiliations:** 1 Department of Medicine, Queen’s University, Kingston, ON K7L 2V6, Canada; 2 Canadian Cancer Trials Group, Queen’s University, Kingston, ON K7L 3N6, Canada; jsleeth@ctg.queensu.ca (J.S.); judy.needham@telus.net (J.N.); mbroekhoven@ctg.queensu.ca (M.B.); MDjurfeldt@ctg.queensu.ca (M.D.); lshepherd@ctg.queensu.ca (L.ES.); bechen@ctg.queensu.ca (B.EC.); jlpater@ctg.queensu.ca (J.LP.); 3 Sunnybrook Health Sciences Centre, Sunnybrook Research Institute, University of Toronto, Toronto, ON M4N 3M5, Canada; Nicole.Mittmann@cadth.ca (N.M.); Matthew.Cheung@sunnybrook.ca (M.CC.); 4 Princess Margaret Cancer Centre, University of Toronto, Toronto, ON M5G 2C1, Canada; Michael.Crump@uhn.ca; 5 Juravinski Cancer Centre/Hamilton Health Sciences, McMaster University, Hamilton, ON L8V 5C2, Canada; meyerr@hhsc.ca

**Keywords:** lymphoma, data linkage, clinical trial

## Abstract

In a prospective study, we sought to determine acceptability of linkage of administrative and clinical trial data among Canadian patients and Research Ethics Boards (REBs). The goal is to develop a more harmonized approach to data, with potential to improve clinical trial conduct through enhanced data quality collected at reduced cost and inconvenience for patients. On completion of the original LY.12 randomized clinical trial in lymphoma (NCT00078949), participants were invited to enrol in the Long-term Innovative Follow-up Extension (LIFE) component. Those consenting to do so provided comprehensive identifying information to facilitate linkage with their administrative data. We prospectively designed a global assessment of this innovative approach to clinical trial follow-up including rates of REB approval and patient consent. The pre-specified benchmark for patient acceptability was 80%. Of 16 REBs who reviewed the research protocol, 14 (89%) provided approval; two in Quebec declined due to small patient numbers. Of 140 patients invited to participate, 115 (82%, 95% CI 76 to 88%) from across 9 Canadian provinces provided consent and their full name, date of birth, health insurance number and postal code to facilitate linkage with their administrative data for long-term follow-up. Linkage of clinical trial and administrative data is feasible and acceptable. Further collaborative work including many stakeholders is required to develop an optimized secure approach to research. A more coordinated national approach to health data could facilitate more rapid testing and identification of new effective treatments across multiple jurisdictions and diseases from diabetes to COVID-19.

## 1. Introduction

Jeff, a 67-year-old Canadian accountant, walks into the sparse clinic room with his wife and well-maintained ring binder; in it is contained carefully filed blood results, an up-to-date medication list, blood pressure readings, and summaries of appointments with all of his health care providers. His electronic health care record holds detailed information about his hospital visits, admissions and treatment at this institution, but lacks many occurring at other hospitals, private laboratories and his family doctor’s practice. His private insurance company has specific carefully reviewed data regarding the prescription medication they have reimbursed, and his time off work they have compensated. His health care payer, the provincial funding agency, maintains records of all health care interactions within their province including diagnostic and therapeutic intervention codes and organized data about symptoms reported at each of his cancer clinic visits. Statistics Canada (StatCan), the national statistical agency, holds data about his birth and important diagnoses occurring when he lived in another province. Jeff’s de-identified information is contained in the secure provincial population health registry. The sponsor of a clinical trial on which he is a consenting participant has meticulously cleaned de-identified data about his illness, treatment response and side effects, as well as additional research results from studies performed on his blood and tissue specimens. All of these carefully curated data sources have unique strengths. The information held across them is complementary, but results in an absurd duplication in effort, as all of these stewards of health data holders do their best to collect accurate information and maintain it securely. Surely, we can do better at integrating all the valuable information separately held on trial participants.

We previously explored probabilistic linkage of clinical trial and administrative data using limited patient identifiers in a retrospective pilot study with the Institute of Clinical Evaluative Sciences (ICES) in Ontario [1]. This provided insufficient information to reliably identify all required individuals in their database. Hence in the prospective Long-term Innovative Follow-up Extension (LIFE) pilot study presented here, we explored deterministic linkage using individual patient identifiers. At the time of embarking on this project, the pervading perception was that this could not be done, patient privacy being a forefront concern. The first objective, reported herein, was to determine acceptability of data linkage among Canadian patients and Research Ethics Boards (REBs). The overall objective is to develop a more rational approach to clinical trial conduct, potentially improving quality and comprehensiveness of data collection at reduced cost and inconvenience for patients. Successfully establishing such an approach would have much broader implications beyond the project presented below.

## 2. Experimental Section

### 2.1. Setting

The Canadian Cancer Trials Group (CCTG) LY.12 randomized phase III trial (NCT00078949) in aggressive lymphoma formed the population basis for this study. Between 2003 and 2011, 619 patients enrolled; of these 530 were from Canada and the remainder accrued from the United States (US), Italy and Australia. The clinical trial met its primary endpoint, demonstrating non-inferiority of rituximab, gemcitabine, dexamethasone and cisplatin (R-GDP) compared with rituximab, dexamethasone, cytarabine and cisplatin (R-DHAP) chemotherapy in patients with relapsed disease prior to autologous stem cell transplantation [2]. Traditional clinical trial follow-up through hospital visits ceased in 2018.

### 2.2. Patients

The LIFE study was added through a protocol amendment approved by Health Canada in December 2016. Following review and approval by REBs, all individuals at participating institutions in Canada who remained alive were invited to participate in the long-term follow-up component and provide their name, date of birth, sex at birth, provincial health insurance number, and postal code for future follow-up through linkage with their administrative records in StatCan and provincial cancer registries. The Ontario Health-Cancer Care Ontario (OH-CCO) registry was selected in this pilot project to be representative of an approach that could be taken across all provinces.

### 2.3. Study Design

**Figure 1:**
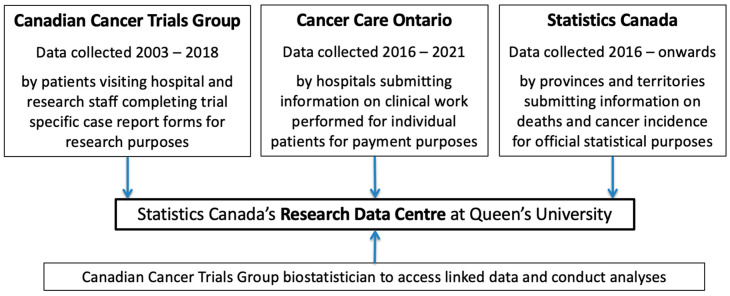
LIFE study design.

The CCTG, StatCan and OH-CCO worked together to develop a process for data transfer, linkage and analysis, complying with regulatory requirements and each organizations’ policies (Figure 1). Data sharing agreements were developed with input from Privacy Officers, REBs and legal counsel.

### 2.4. Data Transfer from Hospitals to the Canadian Cancer Trials Group

Potentially eligible patients were invited to participate by research staff at their local hospital. For those who provided consent, their identifying data were directly entered by site staff onto a web-based application. This application is housed at the CCTG (Kingston, ON, Canada) on a dedicated collection host with 2 state of the art firewalls segmenting it separately from other operational hosts. The security enhanced application is dedicated to this study and function and is restricted to only those investigators and staff participating on this trial. All data are encrypted in transit with Secure Sockets Layer (SSL). Previously collected de-identified clinical trial outcome data, and more recently provided personally identifying information, are held on two separate servers. Only authorized programmers can perform data linkages using the pseudonymous CCTG unique patient identifier code; this unique identifier permits record linkage but in itself has no meaning attached to the individual.

### 2.5. Data Transfer from Ontario Health-Cancer Care Ontario to the Canadian Cancer Trials Group

CCTG securely shared personal identifiers of consenting participants with OH-CCO in order for them to be identified from their registry. OH-CCO will send additional clinical information on cancer recurrence, second cancers and vital status during the follow-up period to CCTG, using the CCTG patient identifier code.

### 2.6. Data Transfer from the Canadian Cancer Trials Group to Statistics Canada

Separately, one file containing patient identifiers (name, date of birth, sex at birth, provincial health insurance number and postal code) and one file containing clinical trial data (disease characteristics, treatment and outcomes) were encrypted and transferred from CCTG to StatCan, through secure electronic means. Data contained within these two files were linked at StatCan through use of the CCTG unique patient identifier code. StatCan used patient identifiers provided to identify each consenting individual in their records, subsequently stripping all patient identifiers. To this linked de-identified data, StatCan can add their additional information on vital status and any occurrence of malignancy reported during the follow-up phase obtained from the Vital Statistics—Death Database (CVSD) and the Canadian Cancer Registry (CCR). This linked dataset will be made available exclusively to the CCTG as deemed employees working within the StatCan’s Research Data Centre (RDC) at Queen’s University (http://www.statcan.gc.ca/eng/rdc/index (accessed date 7 March 2021)). No other researchers other than those affiliated with the CCTG research team will have access to these data. Within the secure RDC environment, the CCTG biostatistician can conduct analyses of the integrated data from all sources. Long-term follow-up will last as long as clinically meaningful to fully understand the benefits and risk of interventions tested in the original clinical trial. This will require additional data linkages as the CVSD and the CCR are annual administrative surveys.

### 2.7. Outcomes

We prospectively designed a global assessment of this innovative approach to clinical trial follow-up including rates of REB approval, patient consent, additional clinical events captured, and the cost of process to ascertain its value. The pre-specified benchmark for patient acceptability was 80%; if ≥80% of those invited agreed to participate, the approach would be considered worthy of further development. We expected that the 95% confidence limits would be less than +/−7.5% of the estimated consent rate. This article focuses on the planned assessment of acceptance rates. The remaining LIFE study endpoints of the economic analysis comparing the cost of the innovative clinical trial follow-up process with that of traditional follow-up through treating institutions, and lymphoma-specific analyses, will be reported at a later date when these data are mature.

## 3. Results

Of the 16 REBs across Canada asked to review the protocol amendment, 14 (89%) approved representing all 9 provinces in which the study was open. The original clinical trial was only open to centers with stem cell transplant programs, hence there were no participating centers in the Territories or Prince Edward Island. Following consideration, two of the REBs in the province of Quebec refused to approve sponsor access to patient identifying information because (i) the committee considered “the need to provide identifying information to the Canadian Cancer Trials Group and to disseminate it to organizations such as StatCan has not been demonstrated” and (ii) only a small number of patients were eligible at their institutions. Helpful discussion ensued between members of these REBs and CCTG in 2017. The REB recognized data linkage may be an appropriate direction for research conduct but wanted to see more data before approving the approach at their institution.

The 140 living participants under follow-up at centers with REB approval who could be reached were invited to enrol in the long-term follow-up study through a combination of in person discussion, telephone discussion and paper mail, depending on center and patient preferences. An optional script was provided to guide research staff leading the informed consent discussion. Of these, 115 (82%) consented, providing identifiers and permission for future data linkage (95% CI: 76% to 88%). Fifteen (18%) of those invited to participate declined; among these, 2 were considered by the consenting research staff to have a language barrier limiting their understanding. Patient acceptability rates varied across centers, ranging between 100% and 0%. Eleven patients, believed to be alive, could not be contacted (Table 1).

There were no statistically significant differences between individuals who provided and did not provide consent to participate (Table 2). Neither age, sex, size of city, timing of enrolment or treatment received on the original LY.12 clinical trial (*p* > 0.5) appeared to influence willingness to participate. In the two centers who approached the greatest number of potential participants, acceptance rates were numerically larger (89% vs. 77%, *p* = 0.07).

## 4. Discussion

We conducted a novel prospective study of the feasibility of clinical trial and administrative data linkage in Canada. The majority of Canadian Research Ethics Boards (14/16 = 89%) approved the research protocol granting permission to approach patients. The majority of Canadian patients invited consented to participate (115/140 = 82%) and provided their full name, date of birth, health insurance number and postal code to facilitate linkage. Acceptance rates varied across centers; one could hypothesize this may be due to chance alone with small patient numbers, regional population differences, or variation in approach to consent by research staff.

These results are similar to previous hypothetical patient surveys. Among 569 individuals with cancer surveyed in Ontario [3], 93% would allow their personal information to be used to match clinical trial with administrative data, and permit long-term research access to the latter. Of 590 individuals with heart disease surveyed in Quebec [4], 80.3% would grant researchers access to health administrative databases with varying levels of comfort for providing identifying information; name (90%), health insurance number (83.9%) and social security number (61.4%). Among 151 individuals who completed a survey conducted by Arthritis Research Canada and the Canadian Skin Patient Alliance, 93.4% felt positively about the use of routinely collected data for health research; de-identification of personal information was the top privacy measure and over half wanted to learn more [5]. In the US, a survey of 677 cancer patients and survivors found that 71% were willing to share de-identified medical data for research purposes; most were motivated by a desire to help other cancer patients [6].

Analysis of routinely collected real-world data is of increasing interest as an alternative approach to clinical trials [7]. Registry-based randomized controlled trials have been successfully used to complete pragmatics studies, particularly in European countries where inhabitants have a unique identification number linked with high-quality national data [8]. Primary and secondary care electronic health records hold potential to support conduct of clinical trials [9,10,11]. However, in a study of clinical trials published in high impact journals, only 15% could feasibly have been replicated using available real-world data sources [12]. Trials of new drugs that had not yet received regulatory approval, educational, behavioural or procedural interventions, medical device trials and those that required symptom reporting were among those in which real-world data alone could not replace a traditional clinical trial [12].

Our recommendation for clinical trial conduct is a hybrid approach, combining the best of rigorous clinical trial conduct with the wealth of routinely collected real-world data. We propose, at the time of enrolment on a clinical trial, consenting individuals are invited to provide their personal identifying information including their provincial health insurance number to facilitate linkage with administrative data sources. For optimal study conduct, linkage would be an integral component of participation; however, this is a complicated issue, and more discussion is needed on the matter of optional versus mandatory provision of identifiers. Traditional clinical trial data collection methods with reporting of outcomes including serious adverse events requiring expedited reporting, specific patient reported symptoms, tumour response rate and other data not otherwise routinely collected, would continue to be gathered through trial-specific case report forms. Outcomes including hospitalization, Emergency Room visits, long-term overall survival, and development of new diseases such as cancer and diabetes, could be obtained with patient consent from real-world data sources. To do so requires highly secure IT platforms and clearly laid out processes for data linkage, with priority placed on data security and ensuring patient privacy rights are upheld. Willingness to collaborate among the many stakeholders including patients, administrative data holders, regulators, privacy officers, REBs, researchers, and funders has been demonstrated by numerous grass roots efforts in Canada (e.g., CanREValue, Health Data Research Network Canada, Pan-Canadian Real-World Health Data Network, Canadian Primary Care Sentinel Surveillance Network) and elsewhere. Internationally and within Canada, approaches to administrative data linkage and analysis vary, with different linkage models at the Manitoba Center for Health Policy, ICES in Ontario, and Population Data BC, for example [13]. A systematic national approach with government support would expedite efficient development and careful evaluation of new approaches enabling the conduct of national multi-center studies in alignment with federal regulations.

Potential benefits of a hybrid approach are (i) the quality of research output may be enhanced with more complete data capture [14], (ii) the cost of conducting research may be reduced allowing a larger number of interventions to be tested within existing budgets [14], (iii) research may be conducted at less inconvenience, cost and risk to patients with reduction in research visits to hospital, and (iv) evaluation of medical products may be expedited bringing proven effective interventions more quickly to the wider population who may benefit. Testing and adoption of any novel approaches must be closely monitored to ensure that health care regulators, policy makers, patients and physicians, continue to receive reliable results on which to base important personal, provincial and national decisions.

A number of limitations provide direction for future collaborative research and planning. The LIFE study sample size is small with 140 patients approached and we did not collect reasons from those who declined participation. Limitations of existing administrative and registry data in Canada include its provincial nature, with challenges in merging data from various sources, of particular importance to national research spanning political boundaries. Delays of up to 2–3 years exist before data in some administrative sources become available; this could pose a major challenge for timely analysis if for example it was being used to inform a primary research outcome of overall survival. Substantial collaborative effort was required to reach agreement aligning with policies and privacy requirements of StatCan, OH-CCO, CCTG and participating hospitals; this would become more complex with wider roll out with inclusion of additional relevant data holders although over time and with increased practice, standardized protocols and agreements could be developed to streamline the process. Our study population consisted of patients treated for aggressive lymphoma; it would be valuable to replicate their findings in a group being treated for less severe illness to see if they would have a similar acceptance rate. The potential tension between patient privacy and enabling high quality rapid research for patient benefit, requires careful consideration with input from many to determine where the optimal balances lie in a given society and time. Successfully securing the funding to support long-term follow-up is challenging.

## 5. Conclusions

The high demonstrated rates of REB approval and patient acceptance in this long-term innovative follow-up study encourages further exploration of a national harmonized approach to data. Such an approach is relevant across geographical boundaries and in multiple diseases from cancer to diabetes to COVID-19. Particularly during a pandemic when individuals’ movements are restricted, hospital staff are strained in providing care to those who need it most, and the need for rapid access to high quality data is foremost, alternative approaches to research merit pursuit [15]. With careful attention to minimizing risk while maximizing benefit, major strides can be taken towards the goal of providing individuals like Jeff with a more coordinated approach to clinical care and research, and the potential to more rapidly test and prove new treatments for the illnesses that afflict them.

## Figures and Tables

**Table 1:** LIFE study patient acceptance rates by center.

Center	Consented to LIFE Study Participation *n* = 115 (%)	Declined LIFE Study Participation	Could Not Be Contacted *n* = 11 (%)
		*n* = 25 * (%)	
Alberta			
Edmonton	1 (100)	0 (0)	0 (0)
British Columbia			
Vancouver	0 (0)	1 (100)	0 (0)
Manitoba			
Winnipeg	16 (89)	2 (11)	0 (0)
New Brunswick			
Moncton	1 (100)	0 (0)	0 (0)
Newfoundland and Labrador			
St John’s	4 (80)	1 (20)	0 (0)
Nova Scotia			
Halifax	9 (64)	3 (21)	2 (14)
Ontario			
Kingston	4 (80)	1 (20)	0 (0)
Hamilton	9 (53)	8 (47)	0 (0)
London	12 (100)	0 (0)	0 (0)
Mississauga	2 (67)	1 (33)	0 (0)
Toronto (Princess Margaret)	34 (77)	4 * (9)	6 (14)
Toronto (Sunnybrook)	4 (44)	3 (33)	2 (22)
Total	65 (72)	17 (19)	8 (9)
Quebec			
Montreal	8 (80)	1 (10)	1 (10)
Quebec City	4 (100)	0 (0)	0 (0)
Total	12 (86)	1 (7)	1 (7)
Saskatchewan			
Regina	2 (100)	0 (0)	0 (0)
Saskatoon	5 (100)	0 (0)	0 (0)
Total	7 (100)	0 (0)	0 (0)

* Of those that are recorded as declined, two were due to a language barrier impeding communication.

**Table 2:** Baseline characteristics among those consenting and not consenting to participate in the Long-term Innovative Follow-up Extension (LIFE) study.

	Consenting	Declining
	N = 115	N = 25
Age (years)		
Mean	52.3	50.9
Median	53.8	54.7
Range	24.6–68.4	26.2–66.5
Sex		
Female	44 (80%)	11 (20%)
Male	71 (84%)	14 (16%)
Year enrolled on clinical trial		
2003–2007	63 (83%)	13 (17%)
2008–2011	52 (81%)	12 (19%)
Assigned arm on clinical trial		
Control (R-DHAP)	58 (84%)	11 (16%)
Experimental (R-GDP)	57 (80%)	14 (20%)
Size of city		
Large: Toronto, Montreal, Vancouver	52 (84%)	10 (16%)
Smaller: Others	63 (81%)	15 (19%)
Number of patients approached at center		
18 or more (2 centers)	52 (84%)	9 (16%)
17 or less	65 (77%)	19 (23%)

## Data Availability

The Canadian Cancer Trials Group is committed to responsible data sharing. CCTG’s data sharing policy is available at www.ctg.queensu.ca (accessed on 20 February 2021). Requests may be directed to datasharing@ctg.queensu.ca.
